# Effects of Preoperative Aspirin on Cardiocerebral and Renal Complications in Non-Emergent Cardiac Surgery Patients: A Sub-Group and Cohort Study

**DOI:** 10.1371/journal.pone.0030094

**Published:** 2012-02-02

**Authors:** Longhui Cao, Scott Silvestry, Ning Zhao, James Diehl, Jianzhong Sun

**Affiliations:** 1 Department of Anesthesiology, Jefferson Medical College, Thomas Jefferson University, Philadelphia, Pennsylvania, United States of America; 2 Division of Cardiothoracic Surgery, Jefferson Medical College, Thomas Jefferson University, Philadelphia, Pennsylvania, United States of America; 3 Anesthesiology Department, Sun Yat-Sen University Cancer Center, Guangzhou, People's Republic of China; 4 Department of Psychiatry, University of Pennsylvania Health System, Philadelphia, Pennsylvania, United States of America; University of Modena and Reggio Emilia, Italy

## Abstract

**Background and Objective:**

Postoperative cardiocerebral and renal complications are a major threat for patients undergoing cardiac surgery. This study was aimed to examine the effect of preoperative aspirin use on patients undergoing cardiac surgery.

**Methods:**

An observational cohort study was performed on consecutive patients (n = 1879) receiving cardiac surgery at this institution. The patients excluded from the study were those with preoperative anticoagulants, unknown aspirin use, or underwent emergent cardiac surgery. Outcome events included were 30-day mortality, renal failure, readmission and a composite outcome - major adverse cardiocerebral events (MACE) that include permanent or transient stroke, coma, perioperative myocardial infarction (MI), heart block and cardiac arrest.

**Results:**

Of all patients, 1145 patients met the inclusion criteria and were divided into two groups: those taking (n = 858) or not taking (n = 287) aspirin within 5 days preceding surgery. Patients with aspirin presented significantly more with history of hypertension, diabetes, peripheral arterial disease, previous MI, angina and older age. With propensity scores adjusted and multivariate logistic regression, however, this study showed that preoperative aspirin therapy (vs. no aspirin) significantly reduced the risk of MACE (8.4% vs. 12.5%, odds ratio [OR] 0.585, 95% CI 0.355–0.964, P = 0.035), postoperative renal failure (2.6% vs. 5.2%, OR 0.438, CI 0.203–0.945, P = 0.035) and dialysis required (0.8% vs. 3.1%, OR 0.230, CI 0.071–0.742, P = 0.014), but did not significantly reduce 30-day mortality (4.1% vs. 5.8%, OR 0.744, CI 0.376–1.472, P = 0.396) nor it increased readmissions in the patients undergoing cardiac surgery.

**Conclusions:**

Preoperative aspirin therapy is associated with a significant decrease in the risk of MACE and renal failure and did not increase readmissions in patients undergoing non-emergent cardiac surgery.

## Introduction

Although tremendous progress has been made in the field of cardiac surgery over the past four decades, major cerebral, cardiac and renal complications associated with cardiac surgery remain common and significant [1]–[3]. According to the Society of Thoracic Surgeons (STS) data reports (2009), the 30-day operative death and major complication rates for valve plus coronary artery bypass graft (CABG) procedure were 6.8% and 30.1%, respectively, including stroke (2.9%), renal failure (9.0%), reoperation (11.9%), prolonged ventilation (21.2%), and sternal infection (0.7%) [3].

Importantly, there is still lacking of an effective clinical therapy to prevent these major cardiocerebral and renal complications. Nonetheless, aspirin as an antiplatelet and antiinflammatory agent has been one of major medicines in prevention and treatment of cardiovascular disease (CVD). Accumulating evidence has demonstrated that aspirin significantly reduces all-cause mortality, MI and stroke in patients with risk of CVD [4]–[7]. Meanwhile, early postoperative aspirin therapy has been applied to improve postoperative outcomes in patients undergoing CABG, including improved graft patency, a reduced risk of death and ischemic complications [8]–[13]. However, it remains to be determined about whether preoperative aspirin therapy can reduce major adverse cardiocerebral (MACE) and renal events in patients undergoing cardiac surgery [14]–[16]

Based on the finding of aspirin's overall beneficial effects in patients with CVD from previous large clinical trials and meta-analysis [4]–[7], we hypothesized that preoperative use of aspirin, mainly through its antiinflammatory and antithrombotic effects, would provide cardiovascular protection against major cardiocerebral and renal complications in patients undergoing cardiac surgery. Thus, the present study aimed to test the overall effects of preoperative aspirin use on cardiocerebral and renal outcomes in patients undergoing non-emergent cardiac surgery.

## Methods

### Study Design

**Figure 1:**
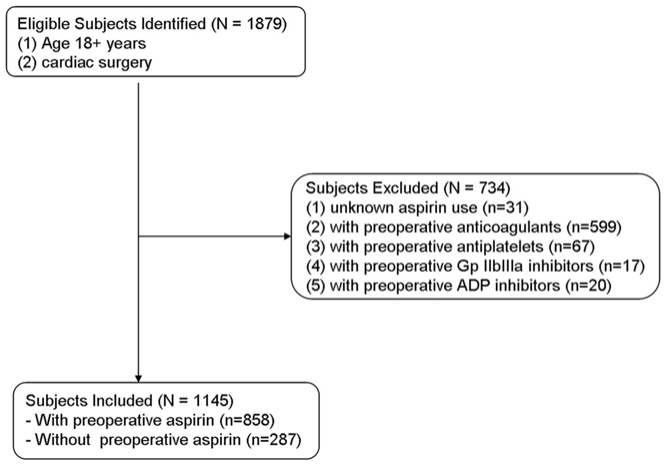
Selection of study sample.

This study was an observational cohort study involving consecutive patients (n = 1879) receiving cardiac surgery (84% patients were for CABG or/and valve surgery) at this university hospital from August 2003 to December 2009. The study was in compliance with Declaration of Helsinki and reviewed and approved by Thomas Jefferson University Institutional Review Board, and individual consent was waived in compliance with the HIPAA regulations and the waiver criteria. The patients excluded from the study were those with preoperative anticoagulants, unknown aspirin use, or underwent emergent cardiac surgery, i.e., the patient's clinical status includes any of the following: ischemic dysfunction, mechanical dysfunction (such as acute evolving MI or shock with circulatory support) or emergent salvage (see details at: http://www.sts.org/documents/pdf/trainingmanuals/Tab9-SectionIOPERATIVE.pdf. [accessed at July 9, 2010]). Of all patients, 1145 patients met the inclusion criteria and were divided into two groups: using (n = 858) or not using (n = 287) preoperative (within 5 days preceding surgery) aspirin (Fig. 1).

### Data Collection

The patient data were collected and organized to follow the template of the STS national database, including demographics, patient history, medical record information, preoperative risk factors, preoperative medications, intraoperative data, postoperative MACE, renal failure and 30-day all cause mortality. Independent investigators prospectively collected the data on each patient during the course of hospitalization for cardiac surgery. Missing data values for dichotomous variables were assigned the most frequent value, while continuous variables were assigned the median value, except for body surface area, which was assigned the sex-specific median value [17]. Preoperative use of aspirin indicates use of aspirin in the patient within 5 days preceding surgery.

MACE included permanent or transient stroke, coma, perioperative MI, heart block and cardiac arrest. Based on the STS national criteria, permanent stroke is defined as a new-onset cerebrovascular accident persisting >24 h; transient stroke as a transient episode of neurological dysfunction caused by focal brain, spinal cord, or retinal ischemia, without acute infarction; coma, the patient had a new postoperative coma that persists for at least 24 hrs secondary to anoxic/ischemic and or/metabolic encephalopathy, thromboembolic event or cerebral bleed; perioperative MI as patient as documented by the following criteria (<24 hours post-op): The CK-MB (or CK if MB not available) must be greater than or equal to 5 times the upper limit of normal, with or without new Q waves present in two or more contiguous ECG leads, no symptoms required; or as documented by at least one of the following criteria (>24 hours post-op): 1) Evolutionary ST- segment elevations, 2) Development of new Q- waves in two or more contiguous ECG leads, 3) New or presumably new LBBB pattern on the ECG, 4) The CK-MB (or CK if MB not available) must be greater than or equal to 3 times the upper limit of normal; heart block as a new heart block requiring the implantation of a permanent pacemaker of any type prior to discharge; postoperative renal failure as acute or worsening renal failure resulting in one or more of the followings: increase in serum creatinine >2.0 mg/dL and 2× most recent preoperative creatinine level over baseline or new requirement for dialysis postoperatively; and readmission as the patient was readmitted as an in-patient within 30-days from the date of initial surgery for any reason. This includes readmissions to acute care, primary care institutions only, not to rehabilitation hospital or nursing home. The remaining definitions are available at http://www.sts.org/documents/pdf/trainingmanuals/adult2.61/V-c-AdultCVDataSpecifications2.61.pdf (accessed at July 27, 2010).

### Statistical Analysis

Continuous and categorical variables were reported as mean ± SD or percentages, and compared with a 2-sample *t* tests or a chi-square test (two tailed), respectively. Univariate and multivariate logistic regression were performed to assess associations of demographic, therapeutic and clinical outcome variables.

As described previously [18], because this was an observational study, a propensity score-adjusted analysis was performed to control for selection bias as result of nonrandom assignment to the two groups. A propensity score was derived, reflecting the probability that a patient would receive preoperative aspirin. This was accomplished by performing a multivariable logistic regression analysis using preoperative aspirin as the dependent variable and entering all baseline (preoperative) variables as in table 1 that clinically would likely affect the probability of using preoperative aspirin.

**Table 1:** Demographic and clinical characteristics.

Characteristics	Aspirin		*P* value
	Yes	No	
	n = 858	n = 287	
Age, yrs	65.3±12.0	59.1±15.3	<0.001
Male gender, %	602(70.2)	167(58.2)	<0.001
Body mass index, kg/m2	29.6±9.3	29.8±15.0	0.774
Past medical history			
Diabetes	300(35.0)	68(23.7)	<0.001
Hypertension	724(84.4)	198(69.0)	<0.001
Smoker	163(19.0)	63(22.0)	0.276
Cerebrovascular disease	116(13.5)	30(10.5)	0.178
Peripheral vascular disease	94(11.0)	12(4.2)	0.001
Chronic lung disease	256(29.8)	78(27.2)	0.391
Family History CAD	500(58.3)	123(42.9)	<0.001
Clinical pattern			
Angina	248(28.9)	50(17.4)	<0.001
Congestive heart failure	104(12.1)	43(15.0)	0.210
Previous MI	222(25.9)	42(14.6)	<0.001
Multiple CAD	661(77.0)	108(37.6)	<0.001
Left main CAD	167(19.5)	18(6.3)	<0.001
Medical therapy			
Beta-blockers	662(77.2)	140(48.8)	<0.001
Diuretics	253(29.5)	80(27.9)	0.603
Digitalis	35(4.1)	11(3.8)	0.854
ACE or ARB Inhibitors	357(41.6)	83(28.9)	<0.001
Perfusion time (min)	104.3±46.9	120.2±56.2	<0.001
Cross-clamp time (min)	82.6±41.3	95.3±49.5	<0.001

Values are n (%) for categorical variables and mean±SD for continuous variables.

In this study, the propensity score was used in regression (covariance) adjustment [19], i.e., using large set of preoperative variables as above to estimate the propensity score, and then the propensity score was subsequently regressed as an independent covariate in the multivariate logistic regression analysis, which was performed by using all relevant variables to identify independent predictors or risk factors for postoperative MACE, renal failure, and mortality.

Potential preoperative confounding factors considered in this analysis were selected on the basis of a literature review, clinical plausibility and variables collected in the database. These variables included (1) demographic characteristics such as age, gender, and body mass index (BMI); (2) patient history such as diabetes, hypertension, peripheral vascular disease, cerebrovascular disease, chronic lung disease, family History of coronary artery disease (CAD); (3) preoperative risk factors such as angina, congestive heart failure, previous MI, multiple CAD, left main CAD, and preoperative medications such as β-blockers, digitalis, diuretics and rennin-angiotensin system inhibitors (RAS inhibitors including angiotensin-converting enzyme [ACE] Inhibitors or angiotensin-II receptor blockers [ARB]) in addition to aspirin and (4) intraoperative factors including perfusion time and cross-clamp time.

Models fit analysis was evaluated with the Hosmer-Lemeshow goodness-of-fit statistic. The C statistic was reported as a measure of predictive power. Results are reported as percentages and odds ratios (OR) and with 95% confidence intervals (CI). All reported p values were 2-sided, and p values<0.05 were considered to be statistically significant. Statistical analysis was performed with SPSS 17.0 software for Windows (SPSS Inc., Chicago, IL).

## Results

### Baseline and intraoperative parameters

Of 1879 patients in the database, 1145 patients met the inclusion criteria and were divided into two groups: using (n = 858) or not using (n = 287) preoperative (within 5 days preceding surgery) aspirin (Fig. 1). Demographic and clinical data of the patients who did and did not receive preoperative aspirin therapy are presented in Table 1. No significant differences were evident between two groups in body mass index (BMI), medical history (smoking, cerebrovascular disease, chronic lung disease), clinical pattern (congestive heart failure, cardiogenic shock), and preoperative medical therapy (digitalis, diuretics use). However, the patients with aspirin presented more with history of hypertension (84.4% vs. 69.0%, P<0.001), diabetes (35.0% vs. 23.7%, P<0.001), peripheral vascular disease (11.0% vs. 4.2%, P<0.001), previous MI (25.9% vs. 14.6%, P<0.001), angina (28.9% vs. 17.4%, P<0.001) and family history of coronary artery disease (CAD) (58.3% vs. 43.9%, P<0.001). And the patients with aspirin also presented more with preoperative using beta-blockers and rennin-angiotensin system (RAS) inhibitors. Meanwhile, the procedural characteristics, including perfusion time (120.2±56.2 vs. 104.3±46.9, P<0.001) and aortic cross-clamp time (95.3±49.5 vs. 82.6±41.3, P<0.001), were significantly longer in the patients without aspirin.

### Postoperative cardiocerebral and renal complications and mortality

**Figure 2:**
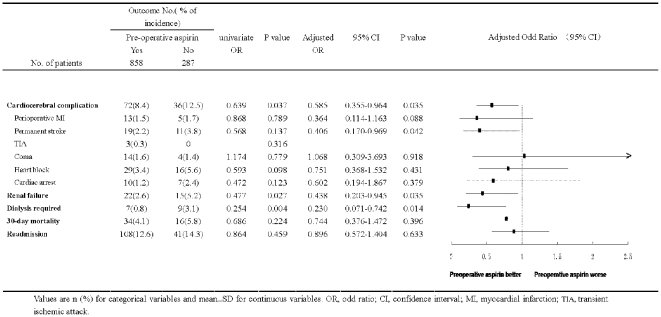
Effects of aspirin on postoperative complications and mortality in patients undergoing cardiac surgery. Values are n (%) for categorical variables and mean±SD for continuous variables. OR, odd ratio; CI, confidence interval; MACE, major adverse cardiocerebral events; MI, myocardial infarction; TIA, transient ischemic attack.

Among 1145 patients undergoing cardiac surgery, a total of 9.5% of all patients experienced at least one of cardiocerebral complications, including permanent or transient stroke, coma, perioperative MI, heart block and cardiac arrest. The incidence of MACE in patients who received preoperative aspirin was 8.4% compared with 12.5% for patients who did not receive aspirin (P = 0.035), indicating preoperative use of aspirin significantly decreased the risk of a composite outcome - cardiocerebral complications (by 33.3%) in patients undergoing cardiac surgery (Fig. 2).

Among other complications, compared with no aspirin preoperatively, preoperative use of aspirin also significantly reduced the risk of postoperative renal failure (2.6% vs. 5.2%, P = 0.035) and dialysis required (0.8% vs. 3.1%, P = 0.014). Importantly there was no difference in incidence of readmission (Fig. 2), which was most due to such as pericardial effusion and/or tamponade, deep stern infection, pneumonia or respiratory complication, arrhythmia and etc, indicating that an obvious increase in postoperative bleeding that needs to be admitted did not occur in patients taking preoperative aspirin.

Overall, the 30-day all cause mortality rate was 50 of 1145 (4.4%). The 30-day mortality was 4.1% for patients with preoperative aspirin and 5.8% for patients without one (P = 0.396). The 30-day mortality rate was 9.3% (10/107) for patients with postoperative cardiocerebral events compared with 4.0% (40/988) for patients without postoperative cardiocerebral events (P = 0.013), indicating postoperative cardiocerebral complications significantly contributing to the death associated with cardiac surgery.

### Independent risk factors for MACE

The unadjusted univariate analysis showed that risk factors related with MACE were age, male sex, diabetes, hypertension, angina, congestive heart failure, multiple CAD, preoperative aspirin, diuretics and digitalis therapy, perfusion time and cross-clamp time (Table 2).

**Table 2:** Univariate Logistic Regression Analysis for Risk Factor Associated with Postoperative Cardiocerebral Events.

Characteristics	Cardiocerebral Events		*P* value
	Yes	No	
	n = 108	n = 1037	
Age, yrs	61.4±14.1	64.0±13.0	0.043
Male gender, %	84(72.5)	685(66.1)	0.014
Body mass index, kg/m2	29.0±7.6	29.7±11.3	0.510
Past medical history			
Diabetes	46(42.6)	322(31.1)	0.015
Hypertension	96(88.9)	826(79.7)	0.021
Smoker	27(25.0)	199(19.2)	0.149
Cerebrovascular disease	12(11.1)	134(12.9)	0.591
Peripheral vascular disease	11(10.2)	95(9.2)	0.727
Chronic lung disease	27(25.0)	307(29.6)	0.316
Family History CAD	50(46.3)	573(55.3)	0.075
Clinical pattern			
Angina	38(35.2)	260(25.1)	0.023
Congestive heart failure	43(39.8)	104(10.0)	<0.001
Previous MI	31(28.7)	233(22.5)	0.143
Multiple CAD	61(56.5)	708(68.3)	0.013
Left main CAD	22(20.4)	163(15.7)	0.211
Medical therapy			
Beta-blockers	82(75.9)	720(69.4)	0.161
Diuretics	49(45.4)	284(27.4)	<0.001
Digitalis	9(8.3)	37(3.6)	0.016
ACE or ARB Inhibitors	47(43.5)	393(37.9)	0.253
Aspirin	72(66.7)	786(75.8)	0.037
Perfusion time (min)	131.4±71.3	105.8±46.3	<0.001
Cross-clamp time (min)	103.2±61.1	84.0±41.2	<0.001

Values are n (%) for categorical variables and mean±SD for continuous variables.

Figure 2 (3 columns on the right) presents the multivariate analysis to assess independent risk factors for postoperative complications, including cardiocerebral (MACE) and renal complications and 30-day all-cause mortality. After adjusting for propensity score and covariates, preoperative aspirin did not show a significant effect on readmission (12.6% vs. 14.3%) and 30-days all cause mortality (4.1% vs. 5.8%), also individual adverse cardiocerebral events including periopeative MI (1.5% vs. 1.7%), coma (1.6% vs. 1.4%), heart block (3.4% vs. 5.6%) and cardiac arrest (1.2% vs. 2.4%).

Using multivariable logistic regression adjusted with propensity scores, however, patients who took preoperative aspirin compared with without one were associated with a significant decrease in the risk of postoperative MACE, permanent stroke, renal failure and dialysis required (Fig. 2).

The multivariate model significantly predicted the occurrence of postoperative cardiocerebral complications (model χ^2^, 90.48; *P*<0.001). The discriminatory ability of the logistic model was acceptable (C statistic, 0.749; 95% CI, 0.697 to 0.801; *P*<0.001). The model was well calibrated among deciles of observed and expected risk (Hosmer-Lemeshow χ^2^, 8.60; *P* = 0.38).

## Discussion

The major findings from this observational cohort study are that preoperative use of aspirin is associated with a significant decrease in the risk of postoperative MACE (8.4% vs. 12.5%), renal failure (2.6% vs. 5.2%) and dialysis required (0.8% vs. 3.1%), meanwhile it is not associated with increased risk of readmissions in patients undergoing non-emergent cardiac surgery. However, preoperative use of aspirin did not show a significant effect on postoperative mortality in this sub-group study.

Cardiocerebral events are still common postoperative complications for patients undergoing cardiac surgery, including stroke (1.4%–4.6%), cardiac arrest (5.0%), MI (3.1%–9.1%) [1]–[3], [20]–[22]. Although there has been lacking of the effective therapy to prevent these complications, several lines of evidence have demonstrated the effectiveness of aspirin, as an antiplatelet and antiinflammatory medicine, in the prevention and treatment of CVD. First, the Antiplatelet Trialists' Collaboration, a meta-analysis, has shown that among the high risk patients for CVD, aspirin significantly reduced rates of MI, stroke and death [5]. Second, in the setting acute MI and stroke, aspirin therapy reduced cardiovascular morbidity and mortality, including recurrent ischemic stroke [23] and myocardial reinfarction [24]. Third, the antiplatelet therapy with aspirin and clopidogrel (plavix) has been recommended to be started before and continuously in percutaneous coronary intervention [25].

In the setting of cardiac surgery, in 2002, Mangano et al [8] in a prospective multicenter study (n = 5065) showed that among patients who received aspirin within 48 hours after revascularization (CABG), subsequent mortality was 1.3%, as compared with 4.0% among those who did not receive aspirin during this period (OR 0.41, 95% CI 0.27–0.62, P<0.001). In addition, aspirin therapy was associated with a 48% reduction in the incidence of MI (2.8% vs. 5.4%, P<0.001), a 50% reduction in the incidence of stroke (1.3% vs. 2.6%, P = 0.01), a 74% reduction in the incidence of renal failure (0.9% vs. 3.4%, P<0.001), and a 62% reduction in the incidence of bowel infarction (0.3% vs. 0.8%, P = 0.01). The risk of hemorrhage, gastritis, infection, or impaired wound healing was not increased with aspirin use (OR for these adverse events, 0.63; 95% CI 0.54 to 0.74).

Although the strong evidence supporting aspirin treatment in the non-surgical setting and even in the surgical setting, such as immediate postoperative use of aspirin in patients undergoing CABG as described above [8], preoperative use of aspirin is still controversial. A major concern of preoperative use of aspirin is its increasing risk of bleeding and transfusion [10], [15]. As a matter of fact, AHA/ACC [26] the Society of Thoracic Surgeons (STS) [27] and the European Association for Cardio-Thoracic Surgery [28] recommended that patients should stop aspirin several days (ranged from 2–10 days) before elective cardiac surgery, mainly due to concerns of perioperative bleeding.

With these controversies, in 2005, Bybee et al [29] performed a retrospective study on preoperative aspirin therapy and postoperative outcomes in patients (n = 1636) undergoing first-time isolated CABG at a single institution. Major findings of this study are 1) preoperative aspirin significantly lowered postoperative in-hospital mortality compared with those not receiving preoperative aspirin (1.7% vs. 4.4%, adjusted OR 0.34, 95% CI 0.15–0.75, P = 0.007). 2) Rates of postoperative cerebrovascular events including cerebral vascular accident or transient ischemic attack were similar between groups (2.7% vs. 3.8%, adjusted OR 0.67, 95% CI 0.32–1.50, P = 0.31), and 3) Preoperative aspirin therapy was not associated with an increased risk of reoperation for bleeding (3.5% vs. 3.4%, *P* = 0.96) or requirement for postoperative blood product transfusion (adjusted OR, 1.17, 95% CI, 0.88–1.54, *P* = 0.28).

Recently, Jacob et al [30] reported in an observational single institution study that among patients undergoing non-emergent isolated CABG, late (within 5 days of the surgery) use of aspirin (vs. discontinued aspirin ≥6 days before surgery) was associated with no significant difference in a composite outcome of in-hospital mortality, MI and stroke (1.8% vs. 1.7%, P = 0.80) and reoperations for bleeding (3.4% vs. 2.4%, P = 0.10) but more intraoperative transfusions (23% vs. 20%, P = 0.03) and postoperative transfusions (30% vs. 26%, P = 0.009). Although the study of Jacob et al showed that preoperative aspirin was associated with a small increase in transfusion requirements (23% vs. 20%), the patients with preoperative aspirin use (vs. nonaspirin) were associated with increased anticoagulant use (49% vs. 30%, *P*<0.0001), which may cause/contribute to the increase in transfusion requirements.

Compared with these earlier studies, this study showed that preoperative use of aspirin was associated with a decrease of a composite outcome - MACE by 33% in patients undergoing cardiac surgery; it was also associated with reduced the risk of postoperative renal failure and dialysis required. The renal protective effect by aspirin was unexpected when starting this study, though a previous report also showed that preoperative aspirin had beneficial effects on renal function in patients with renal insufficiency undergoing CABG [31]. Potential mechanism(s) for this renoprotection remains to be investigated and probably are more related to anti-inflammatory (than anti-platelet) effects of aspirin in the setting of cardiac surgery.

The present study did show a trend to decrease the death (4.1% vs. 5.8%; OR 0.744, 95% CI 0.376–1.472 *P* = 0.396). The authors recognized, however, that a sample size larger than the present one would be needed to determine the effect of aspirin on postoperative mortality (to detect a statistical difference), which has been demonstrated in our recent study [32]. Noticeably, the patients with preoperative aspirin were older and sicker, such as more with history of hypertension, diabetes, peripheral vascular disease, previous MI, angina, left main and multiple CAD (as seen in Table 1). Nevertheless, this study provided additional evidence (to a recent study [32]) that aspirin protects the heart, brain and kidneys against those major risk factors in a sub-group (non-emergent) of patients, indicating its efficacy and potential application to these high-risk patients.

*Limitations of this study*. This is an observational cohort study. Although multivariate regression in combination with the propensity score adjustment was used in this study to reduce overt biases, the potential flaws of a non-randomized study may remain. Second, this is a separate and sub-group study on preoperative aspirin and cardiac surgery, which excluded the patients undergoing emergent cardiac surgery; multicenter, larger (than the present one in the sample size) and cohort studies are needed to investigate this subject step-by-step, as showed in our recent study [32]. Third, cardiac surgery patients share the common risk of postoperative complications involving the brain, heart and kidneys, despite of undergoing different cardiac surgeries. While aspirin, mainly through its antiinflammatory and antithrombotic effects, may break common final pathways responsible for these complications. Thus, although this study provided an overall analysis on effects of preoperative aspirin on outcomes in patients undergoing cardiac surgery (mainly CABG and/or valve surgery), further studies to dissect different types of cardiac surgery (CABG, valve, emergent or elective alone or/and combinations) are needed and probably would provide more detail information about aspirin and cardiac surgery. As indicated before, nonetheless, “the overall result of the clinical study (trial) is usually a better guide to the direction of effect in subgroups than the apparent effect observed from the individual subgroups” [33]. Finally, this study did not provide detailed information about perioperative bleeding, and further studies are still needed to examine this potential side effect carefully (on a case by case base).

*In conclusion*, the results of this study showed that preoperative use of aspirin is associated with a significant decrease in the risk of MACE and renal complications in patients undergoing non-emergent cardiac surgery; these beneficial effects were not associated with increased risk of readmissions. Further clinical studies including randomized or (large) observational studies are needed to elucidate the role of preoperative aspirin in cardiac surgery.
